# Uterine Hæmatocele

**Published:** 1862-02

**Authors:** 


### UTERINE HÆMATOCELE.

(*Continued from page* 44.)

Reprinted from West on the *Diseases of Women.*

There are *four conditions* with which this *uterine hæmatocele* may be *confounded:* viz: extra-uterine pregnancy, retroversion of the pregnant uterus, inflammation of the cellular tissue between the uterus and rectum, and ovarian tumor; and the points of similarity between each of these are quite sufficient to lead very readily into error. The suppression of the menses, the abdominal or pelvic discomfort, and the sense of bearing down backwards, are symptoms common to effusion of blood behind the uterus, and to an extra-uterine fœtation between the second and fourth months; while the general contour of the tumor is very similur in the two cases, and there is the same remarkable pulsation of the vessels distributed to it in both. The attacks of pain in the extra-uterine fœtation are, however, usually more intense and more paroxysmal, while the discomfort in the intervals is less; the sanguineous discharge is absent, and the uterus, if examined with the sound, is ascertained to be increased in size; and even without it the condition of the os uteri and portio vaginalis of the cervix, with the puffy lips, the closed orifice, and the swollen tissue, differs widely from the completely undeveloped state of those parts in cases of hemorrhage about the womb.

The effusion when considerable may cause, as it did in the case which I have related, complete retroversion of the womb, a condition which, when associated as it is sometimes with suppression of the menses for two or three months, may raise the suspicion of pregnancy, and lead to the tumor being taken for the fundus of the enlarged and misplaced uterus. Professor Crédé, of Berlin, relates an instance in which these very circumstances led him for a moment into error, and in which he endeavored vainly to replace what he supposed to be the pregnant and retroverted womb. Further observation soon led him right, and the same considerations as rectified his diagnosis may keep us from error. The cervix and os uteri presented none of the changes of pregnancy; the bladder was not affected; and the uterine sound, which entered readily in the natural direction, could not be turned round with its concavity backwards, nor be made to enter the tumor, intimately though it seemed connected with the womb.

The characters of the tŭmor in cases of inflammation of the uterine cellular tissue very closely resemble those of uterine hæmotacele, and the history and symptoms present a very marked analogy in the two affections. There are, however, some points of difference between them which are generally sufficiently marked to preserve the attentive observer from error. Pelvic abscess is very generally the consequence of delivery or of abortion, while it is scarcely ever associated with any other form of menstrual disorder than its *sudden* suppression; the inflammatory symptoms developing themselves directly out of that accident. Uterine hæmatocele, on the contrary, is seldom the *immediate* consequence of a single suppression of menstruation; it is not unfrequently preceded by menorrhagia, and is often accompanied, at any rate for a time, by a copious sanguineous discharge, a symptom which never attends upon inflammation of the cellular tissue in the vicinity of the uterus. Moreover, the tumor consequent on inflammation is at first very firm and resistant, and becomes soft only by degrees with the advance of suppuration. The tumor of uterine hæmatocele, on the contrary, is soft at first, and becomes more resistant in time, as the fluid elements of the blood are partially removed, while at no period are there the same thickening and induration about it which are so remarkable in that part of the vaginal wall adjacent to any collection of matter.

Ovarian cysts occupy, when small, the same situation as uterine hæmatocele; they are not, however, so sudden in their occurrence, nor so rapid in their increase; while, though their development is often associated with menstrual irregularity, they are not attended by any constant sanguineous discharge. The ovarian tumors, too, do not descend equally low into the recto-vaginal pouch, and consequently do not produce the same difficulty in defecation, while, further, they are not so intimately connected with the uterine wall, and the womb can usually, by means of the sound, be completely isolated from the adjacent swelling.

The number of instances of this affection hitherto observed is scarcely sufficient to enable us to determine accurately the degree of danger attaching to it, any more than the comparative frequency of the intra and extra-peritoneal variety of the hemorrhage. Including the four cases which came under my own observation, I can find some account, though often very meagre, of 41 instances of uterine hæmatocele, 33 of which terminated in recovery, 8 in death; or, in other words, the deaths were in the proportion of 19.5 per cent. of the total number of cases. In one of the fatal cases death took place from phthisis, and was therefore the indirect rather than the immediate result of the affection; twice it resulted from loss of blood, which, however, was in one of the instances due to the accidental wounding of a vessel of the cervix uteri; once it took place under the symptoms of pyæmia, and in the remaining four instances was produced by peritonitis of a rather chronic kind; the patient surviving a month in one case, forty-five days in another, four and a half months in the third, and seven and a half months in the last.

There can, I apprehend, be little doubt but that the real fatality of this affection is considerably less than would appear from our present imperfect data. On the one hand, some of the cases, such as that of M. Bernutz and of Piogey, have been reported as pathological rarities; and, on the other, many which have had a favorable issue have been unrecorded. Many, too, have unquestionably passed unrecognized; for the disposition to the spontaneous absorption of the effused blood, unless the quantity poured out has been enormous, seems to be very great, and menstrual disorder and abdominal pain have probably often passed away without a suspicion having arisen of their connection with hemorrhage around the uterus, or into the cavity of the peritoneum. Still, every allowance being made for the influence of these circumstances, uterine hæmotecele must, I imagine, be always regarded as an accident of a much graver kind than mere inflammation of the cellular tissue in the neighborhood of the uterus, or of its appendages.

In the *treatment* of this affection two different modes of procedure have been advocated, of which one is the expectant plan; while early interference and complete evacuation of the sac are the principles of the other. The statistics of the two methods yield the following results:—

| | | | | | |
|---|---|---|---|---|---|
| Treated on the expectant plan | 14 | Recovered | 11 | Died | 3 |
| " by puncture . . . | 27 | " | 22 | " | 5 |
| | — | | — | | — |
| | 41 | | 33 | | 8 |

but from such slender data I should hesitate to draw any conclusion. I imagine, indeed, that neither plan can be regarded as absolutely the best, but that the special circumstances of each case must guide us. In three of my cases, that alone excepted in which the effusion had already become a chronic evil, the puncture was followed by peritoneal inflammation, which was once of great severity; and the existence of an opening in the vagina did not in that instance prevent the establishment of a communication with the bowels and the discharge of a large quantity of blood per anum. In some instances, too, the fibrin of the blood forms, by its coagulation, a thick layer within the sac, and prevents the escape of the fluid contents after puncture with the trocar; while the enlarging the opening with a bistoury seems to be free neither from the dangers of hemorrhage on the one side, nor from those of inflammation of the cyst on the other. The complete emptying of the cyst, its subsequent washing out with water, and the injection of a solution of iodine into it, as practised by M. Velpeau and advocated by M. Robert, appear to me hazardous proceedings, except when resorted to quite in the chronic state of the affection, when all disposition to hemorrhage has ceased, and the susceptibilities of the cyst wall have become blunted by the lapse of time.

In the earlier stages of the affection, absolute rest, local depletion, and the ministering to each symptom as it occurs, are the indications which we should endeavor to fulfil; while the presence of a tumor even of considerable dimensions, or even its increase to some extent after its first discovery should not, I venture to think, lead us to puncture it, apart from some very serious ill, or suffering clearly attributable to it. In the event of puncture being obviously necessary, a Pouteau's trocar would appear to be the safest and most manageable instrument to employ, and was used in all of my cases. In none of these, however, it must be admitted, was the escape of the blood immediate; but I should imagine that the use of a curved trocar and canula of the thickness of one's finger, such as I have employed to puncture ovarian cysts, per vaginam, would obviate the inconvenience with less risk would be incurred by the use of the knife. After puncture, the great hazard seems to be that of the supervention of inflammation, and my own experience leads me to regard this as very considerable, though it was controlled in each instance by active treatment.

Further experience may very possibly modify some of the views I have just now expressed, and may show that the balance inclines greatly in very early interference in these cases. I may just add, however, that the opinions of M. Nélaton appear to lean even more decidedly than at first they did towards the adoption of an expectant plan of treatment, and to leaving to nature alone the removal of the blood, even though poured out in great abundance.

